# CD64 as a potential biomarker in septic arthritis

**DOI:** 10.1186/1471-2334-13-278

**Published:** 2013-06-19

**Authors:** Oddvar Oppegaard, Brita Skodvin, Anne-Kristine Halse, Nina Langeland

**Affiliations:** 1 Department of Medicine, Haukeland University Hospital, Bergen, Norway; 2 Department of Rheumatology, Haukeland University Hospital, Bergen, Norway; 3 Department of Clinical Science, University of Bergen, Bergen, Norway

**Keywords:** CD64, Procalcitonin, Septic arthritis, Biomarker

## Abstract

**Background:**

Traditional inflammatory markers are generally unhelpful in discerning septic arthritis from inflammatory joint disease due to their lack of specificity. We wished to explore the discriminatory power of the novel inflammatory marker, Fc-gamma-receptor type 1, CD64, in patients presenting with acute arthritis.

**Methods:**

Patients were recruited prospectively in the time period June 2009 to December 2011. Thirty-six patients presenting with an acute flare of chronic rheumatic arthritis, 31 with crystal-induced arthritis and 23 with septic arthritis were included. Traditional inflammatory markers, CD64 and procalcitonin (PCT) were measured and their diagnostic abilities were compared.

**Results:**

CD64 and PCT both demonstrated a specificity of 98%, but poor sensitivities of 59% and 52%, respectively. White blood cell count (WBC), and erythrocyte sedimentation rate (ESR) did not have significant discriminatory power, while C-reactive protein (CRP) proved to have the best diagnostic accuracy as measured by area under the ROC curve (AUC 0.92, 95% confidence-interval 0.87-0.98). Subgroup analysis excluding patients with septic arthritis without concurrent bacteremia, and likewise exclusion of the patients with septic arthritis caused by coagulase negative staphylococci, both improved the diagnostic accuracy of CD64 and PCT, but not of WBC and CRP.

**Conclusions:**

CD64 and PCT are highly specific for infectious disease, but they predominantly measure bacteremia. Their use in hospital practice has yet to be defined, and especially so in localized infections.

## Background

Untreated, septic arthritis has a significant morbidity and mortality [1]. Early recognition is important, as prompt antimicrobial treatment and synovial irrigation reduces the risk of joint destruction. The clinical features, however, are indistinguishable from many other causes of arthritis, especially crystal-induced arthritis (CIA). The diagnosis of septic arthritis depends largely on positive microbiological cultures, but the technique is time-consuming and often delays the final diagnosis by 2–3 days. Furthermore antimicrobial agents administered prior to admission to hospital reduce the diagnostic sensitivity, frequently producing falsely negative results. Traditional inflammatory markers such as white blood cell count (WBC), erythrocyte sedimentation rate (ESR) and C-reactive protein (CRP) are generally unhelpful due to their lack of specificity [2]. These markers are often elevated regardless of whether the inflammatory stimulus is trauma, rheumatic disorders or infectious disease [3].

During the last decade, CD64 has been proposed as a novel and more specific parameter of infection [4]. CD64, also called fc-gamma-receptor type 1, is an integral membrane protein of white blood cells [5]. It interacts with immunoglobulin G with high affinity, and is important for effective phagocytosis of microbial components and immune complexes. The receptor is constitutively expressed on the surface of monocytes, macrophages and eosinophil granulocytes. It is up-regulated on neutrophils as a physiological response to microbial wall components such as lipopolysaccharide, complement split products, as well as cytokines (interferon gamma and granulocyte colony stimulating factor) [6]. The response time is estimated to 4–6 hours [7], making it more versatile than CRP and SR in acutely ill patients.

It has previously been reported that the quantitative measurement of CD64 expression on the surface of neutrophils is a sensitive and specific marker of systemic infection, even in patients with systemic inflammatory diseases [8-11]. We wished to explore the discriminatory power of CD64 in patients presenting with acute arthritis. Comparison was conducted with traditional markers (WBC, SR and CRP) as well as another relatively novel inflammatory marker, procalcitonin (PCT). We included a negative and positive control group to strengthen the foundation for selecting appropriate cut-off values.

## Methods

### Study setting

Haukeland University Hospital, Bergen, Norway, is the referral hospital for a population of about 1.1 million inhabitants and also a local hospital for approximately 350.000 people.

### Patient selection

Patients were recruited prospectively from the hospital in the time period June 2009 to December 2011, inclusion was performed after written informed consent. Four groups of patients were included:

Group 1 (Negative controls) consisted of presumably healthy blood-donors.

Group 2 (Positive controls) consisted of patients admitted to the Emergency room with clinically suspected upper urinary tract infection (UTI), defined by a triad of current urinary tract symptoms (pollakiuria, dysuria, localized bladder/kidney pain, or urine retention), history of fever within the last 2 days and positive urine- and/or blood-culture. Patients with concurrent infectious disease of other etiology were excluded.

Group 3 consisted of patients with established chronic rheumatic arthritis (RA, psoriasis arthritis, oligoarthritis, spondylarthritis or SLE) presenting at the hospital’s Rheumatology outpatient clinic with an acute disease flare as assessed by a skilled rheumatologist (AKH). Infectious etiology was excluded by culture where indicated.

Group 4 consisted of consecutive patients admitted to the hospital with the clinical diagnosis of acute arthritis. Cases were defined by a clinical triade of localized joint pain, local inflammation (rubor, calor, tumor, dolor) and pain provoked by passive joint movement. They were later sub grouped according to final diagnosis as septic arthritis (defined as positive blood-/synovial fluid culture and clinically assessed as infectious etiology by infectious disease specialist), crystal-induced arthritis (defined as presence of urate- or pyrophosphate-crystals in synovial joint-fluid and negative cultures) or other/unknown diagnosis.

### Laboratory investigations

Blood was drawn on admission. CD64 expression was measured by flow cytometry within 48 hours of blood sampling using the commercially available Leuko64TM-kit (Trillium Diagnostics, Brewer, ME, USA) containing calibrated fluorescent beads and antibodies to CD64. The CD64 index was reported using a lot-specific Leuko64 Quanti-CALCTM automated software (Trillium Diagnostics), which calculated the ratio of the mean fluorescent intensity of the examined cell-population to that of the beads. Procalcitonin was measured by the immunological method electrochemiluminescence (Roche/Hitachi MODULAR E170), and CRP by immunoturbidimetric method (Roche/Hitachi MODULAR P). SR and WBC were analyzed by standard methods by our routine laboratory. Arthrocentesis was performed in all patients in group 3 and 4. Synovial fluid samples were cultured for 2 days from native joints and 5 days from prosthetic joints. Negative cultures were further examined by 16sRNA PCR where clinically indicated. The samples were also examined with acridin-orange and gram-stain by a specialist in microbiology, and with polarized light microscopy by a rheumatologist. CD64 expression in synovial fluid was measured by previously described methods, but too few samples were analyzed to permit statistical analysis.

### Statistical analysis

The data are presented as medians and interquartile ranges (IQRs). Subgroups were compared using nonparametric tests (Mann–Whitney *U*-test). Statistical significance was considered at p < 0.05. Receiver-operating characteristics (ROC) curves were created for CD64-index, procalcitonin, CRP and WBC, and were used to establish optimal cut-off values. They also permitted calculation of sensitivity, specificity and area under the ROC curve (AUC). The SPSS software version 20.0 for windows (SPSS Science, Chicago, IL, USA) was used for statistical analysis.

### Ethical considerations

The project was approved by the Regional Ethic committee for Medical research in Western Norway prior to the commencement of the study.

## Results

Group 1 (blood-donors) included 25 participants. Baseline characteristics/demographics for all groups are presented in Table 1.

**Table 1:** The baseline characteristics and measured parameters of the study subjects

	**Blood donor**	**UTI**	**FRA**	**CIA**	**SA**
Cases	25	27	36	31	23
Age	40 (29–57)	66 (29–79)	48 (35–65)	76 (57–87)	60 (47–72)
Sex M/F	17/8	15/12	14/22	21/10	16/7
CD64-index	0.6 (0.5–0.8)	4.9 (2.5–7.8)	1.0 (1.0–1.2)	1.4 (0.9–1.9)	2.3 (0.8–9.3)
PCT (mg/L)	ND	1.29 (0.26–4.42)	0.10 (0.10–0.10)	0.11 (0.10–0.18)	1.27 (0.14–4.41)
CRP (mg/L)	ND	123 (84–235)	23 (8–80)	84 (51–162)	239 (172–308)
WBC (x10^9^/L)	5.3 (4.0-6.3)	16.4 (10.6–18.8)	8.2 (6.8–10.5)	9.8 (8.4–12.1)	11.7 (7.8–13.4)
ESR (mm/t)	ND	55 (36–68)	34 (20–63)	52 (41–71)	77 (63–104)

Data presented as median (inner quartile range). *UTI* urinary tract infection, *FRA* flare of rheumatic arthritis, *CIA* crystal induced arthritis, *SA* septic arthritis, *M* male, *F* female, *PCT* procalcitonin, *CRP* C-reactive protein, *WBC* white blood cell count, *ESR* erythrocyte sedimentation rate, *ND* no data.

In group 2 (Upper urinary tract infection, UTI), 34 patients were assessed for participation. Seven were excluded (two with pneumonia, five with polymicrobial urinary culture). Of the 27 included, nine had positive blood culture, 26 had positive urinary culture. *E. coli* was the most frequent microbe encountered (59%), followed by Klebsiella species (11%) and Enterococcus species (11%). For two patients CD64-analysis was lacking.

In group 3 (Flare of rheumatic arthritis, FRA), 36 patients were included, eight lacked CD64-analysis. The majority of patients suffered from rheumatoid arthritis (39%) and psoriatic arthritis (36%), while the rest consisted of oligo-/polyarthritis (14%) and reactive arthritis (11%).

In group 4 (acute arthritis), 67 patients with acute arthritis were assessed for participation, nine were excluded due to uncertain final diagnosis. Four patients presented with acute arthritis but had spontaneous recovery without the administration of antibiotics. Although no firm diagnosis could be established, they were considered to be of non-bacterial etiology and grouped separately. When calculating the inflammatory markers diagnostic accuracy in discerning infectious from non-infectious etiology, they were grouped with the non-infectious cases (FRA and CIA). Twenty-three had culture-proven septic arthritis, whereof seven were related to prosthetic joints. The characteristics of the septic arthritis cases are presented in Table 2. Twelve had positive blood cultures, while 21 had positive joint fluid cultures. None were diagnosed by 16sRNA-PCR or microscopy alone. The microbial etiology comprised S.aureus (44%), group G streptococci (18%), coagulase negative staphylococci (26%), E.faecalis (4%), Bordetella holmesii (4%) and S.pneumoniae (4%). Six inclusions lacked CD64-analysis. Thirty-one patients were found to have crystal-induced arthritis by examination of joint fluid by polarized light microscopy (15 gout and 16 pseudo gout). Six of these lacked CD64 and one lacked procalcitonin analysis.

**Table 2:** Characteristics of septic arthritis cases

	**NJ/ PJ**	**Microbe**	**Joint culture**	**Blood culture**	**CD64 index**	**PCT**	**CRP**	**WBC**	**Joint**	**Dur. of sympt.**	**AB prior to adm**
**1**	NJ	GGS	+	+	3.2	2.54	239	16.0	Knee	4 d	Yes
**2**	NJ	GGS	+	+	7.3	16.20	219	12.1	Wrist	2 d	No
**3**	NJ	GGS	+	+	ND	1.66	286	16.9	Hip	6 d	No
**4**	NJ	GGS	+	-	ND	5.75	84	13.4	Shoulder	1 d	No
**5**	NJ	S.pneumoniae	+	+	2.4	0.25	273	9.1	Ankle	9 d	Yes
**6**	NJ	E.faecalis	+	-	2.3	1.28	266	9.3	Knee	4 d	Yes
**7**	NJ	S.aureus	-	+	ND	24.30	148	9.6	Shoulder	1 d	No
**8**	NJ	S.aureus	+	+	13.0	2.98	308	29.4	Knee	3 d	No
**9**	NJ	S.aureus	+	+	17.0	2.14	379	6.1	Shoulder	7 d	No
**10**	NJ	S.aureus	+	+	ND	25.30	438	12.3	Shoulder	5 d	No
**11**	NJ	S.aureus	+	-	ND	<0.10	81	6.7	Knee	10 d	No
**12**	NJ	S.aureus	+	-	0.8	<0.10	158	12.9	Ankle	3 d	No
**13**	NJ	S.aureus	+	-	ND	0.13	123	11.8	Knee	13 d	Yes
**14**	NJ	S.lugdunensis	+	-	2.0	0.17	303	8.5	Knee	2 d	No
**15**	NJ	S.lugdunensis	+	-	0.6	0.14	220	7.6	Knee	4 d	No
**16**	NJ	S.lugdunensis	+	-	0.7	0.32	344	11.7	Knee	7 d	No
**17**	PJ^1^	S.epidermidis	+	-	0.7	<0.1	247	7.8	Knee	4 d	No
**18**	PJ^1^	S.capitis	+	-	0.6	<0.10	183	10.2	Knee	3 d	No
**19**	PJ^1^	S.capitis	+	-	0.8	0.24	199	6.5	Knee	7 d	Yes
**20**	PJ^1^	S.aureus	+	+	2.7	4.41	232	6.4	Hip	3 d	No
**21**	PJ^1^	S.aureus	+	+	2.3	0.35	172	14.4	Knee	1 d	No
**22**	PJ^2^	S.aureus	+	+	16.3	9.11	319	12.8	Hip	6 d	No
**23**	PJ^2^	B.holmesii	-	+	11.2	1.27	335	14.0	Hip	3 d	No

*NJ* native joint, *PJ* prosthetic joint, ^1^Early prosthetic infection (< 3 months); ^2^Late prosthetic infection (>12 months); *PCT* procalcitonin, *CRP* C-reactive protein, *WBC* white blood count, Dur. of sympt, duration of symptoms prior to admission in days; AB prior to adm., antibiotics administered prior to admission; *GGS* group G streptococci, *ND* no data.

**Figure 1:**
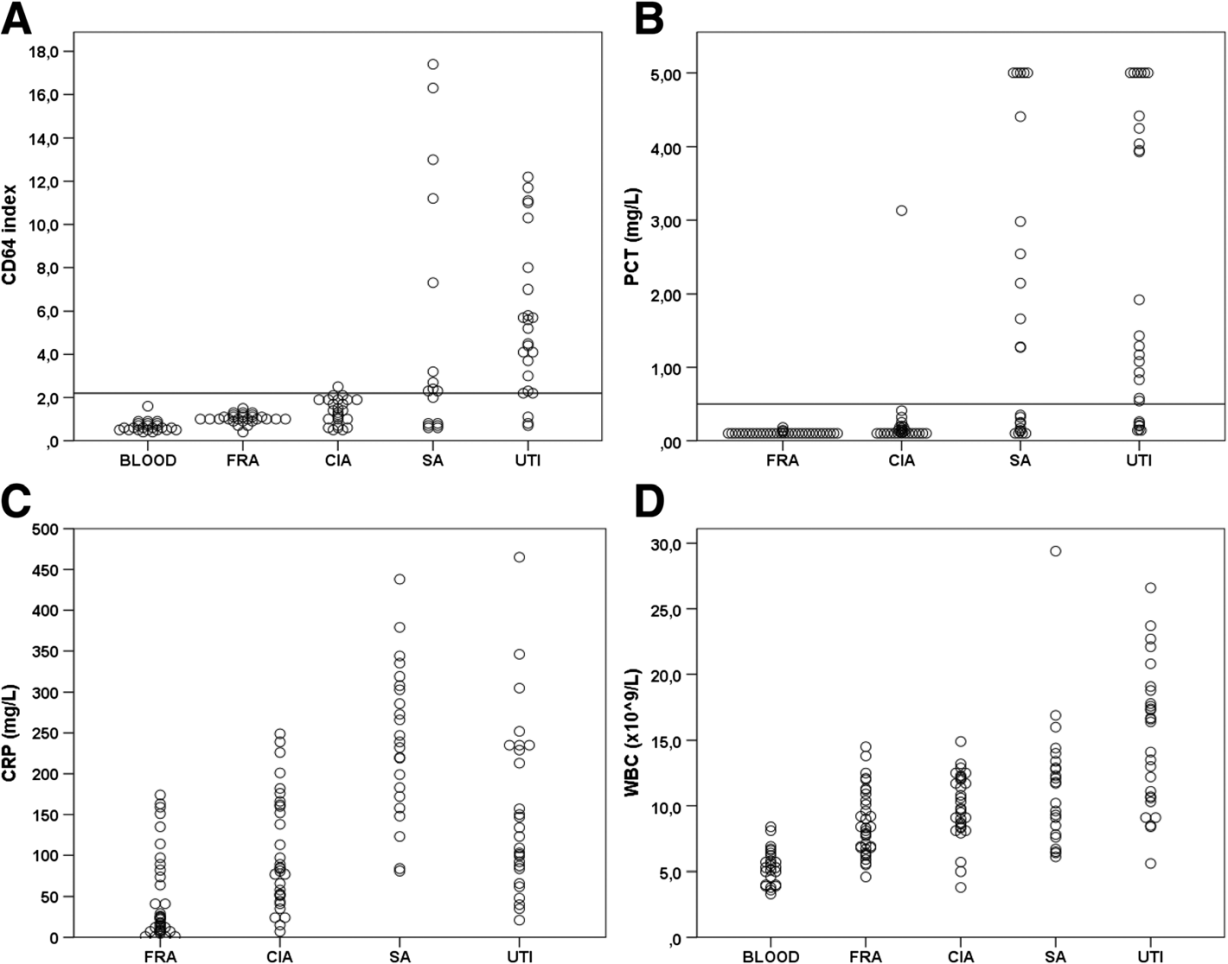
**Serum concentrations of the inflammatory markers.** Serum concentrations of CD64 (**A**), procalcitonin (**B**), CRP (**C**) and white blood count (**D**) among healthy blood donors (BLOOD), patients with flare of rheumatic arthritis (FRA), crystal-induced arthritis (CIA), septic arthritis (SA) and urinary tract infection (UTI). Horizontal lines indicate cut-off values for CD64 (**A**) and PCT (**B**), derived from ROC curve analysis. PCT values above 5 mg/L are plotted as 5 mg/L.

The results of the inflammatory markers are presented in Figure 1. The CD64-index among blood donors showed a median of 0.6 (IQR 0.5-0.8), urinary tract infection (UTI) 4.9 (2.5-7.8), flare of rheumatic arthritis (FRA) 1.0 (1.0-1.2), crystal-induced arthritis (CIA) 1.4 (0.9-1.9) and septic arthritis (SA) 2.3 (0.8-9.3). There were significant differences between SA and CIA (p = 0.03), and between SA and FRA (p = 0.001). The PCT among UTI patients showed a median of 1.29 mg/L (IQR 0.26-4.42), FRA 0.10 (0.10-0.10), CIA 0.11 (0.10-0.18) and SA 1.27 (0.14-4.41). There was a significant difference between SA and CIA (p < 0.001) and between SA and FRA (p < 0.001). The CRP-level among UTI patients showed a median of 123 mg/L (IQR 84–235), FRA 23 (8–80), CIA 84 (51–162) and SA 239 (172–308). Significant differences were found between SA and CIA (p < 0.001), and between SA and FRA (p < 0.001). There were no significant differences found in the WBC and ESR-levels between SA and CIA (both p = 0.3).

ROC-curves were constructed to assess optimal cut-off points and compare diagnostic reliability. When compared by AUC, CRP (AUC 0.92, 95% confidence-interval 0.87-0.98) was found to be the most reliable marker for discrimination between infectious arthritis (SA) and non-infectious arthritis (FRA and CIA) (Table 3). It was followed by PCT (AUC 0.85 (0.74-0.96)) and CD64 (AUC 0.69 (0.51-0.88)), while WBC did not have significant discriminatory power. The ROC curves indicated an optimal cut-off-point for CD64 index at 2.2 and PCT at 0.50 mg/L. This yields a sensitivity of 59% and 52%, and a specificity of 98% and 98% respectively (Table 3).

**Table 3:** Diagnostic abilities of CD64, PCT and CRP in discerning septic arthritis from non-infectious arthritis

	**Sensitivity**	**Specificity**	**AUC**	AUC*
CD64 (cutoff 2.2)	59%	98%	0.69 (0.51–0.88)	0.92 (0.78–1.00)
PCT (cutoff 0.50)	52%	98%	0.85 (0.74–0.96)	0.90 (0.79–1.00)
CRP (cutoff 120)	91%	76%	0.92 (0.87–0.98)	0.91 (0.84–0.98)
CRP (cutoff 240)	48%	98%		

PCT in mg/L; CRP in mg/L; *AUC* area under the curve (95% confidence-interval), AUC*, area under the curve when excluding septic arthritis cases caused by coagulase negative staphylococci.

**Figure 2:**
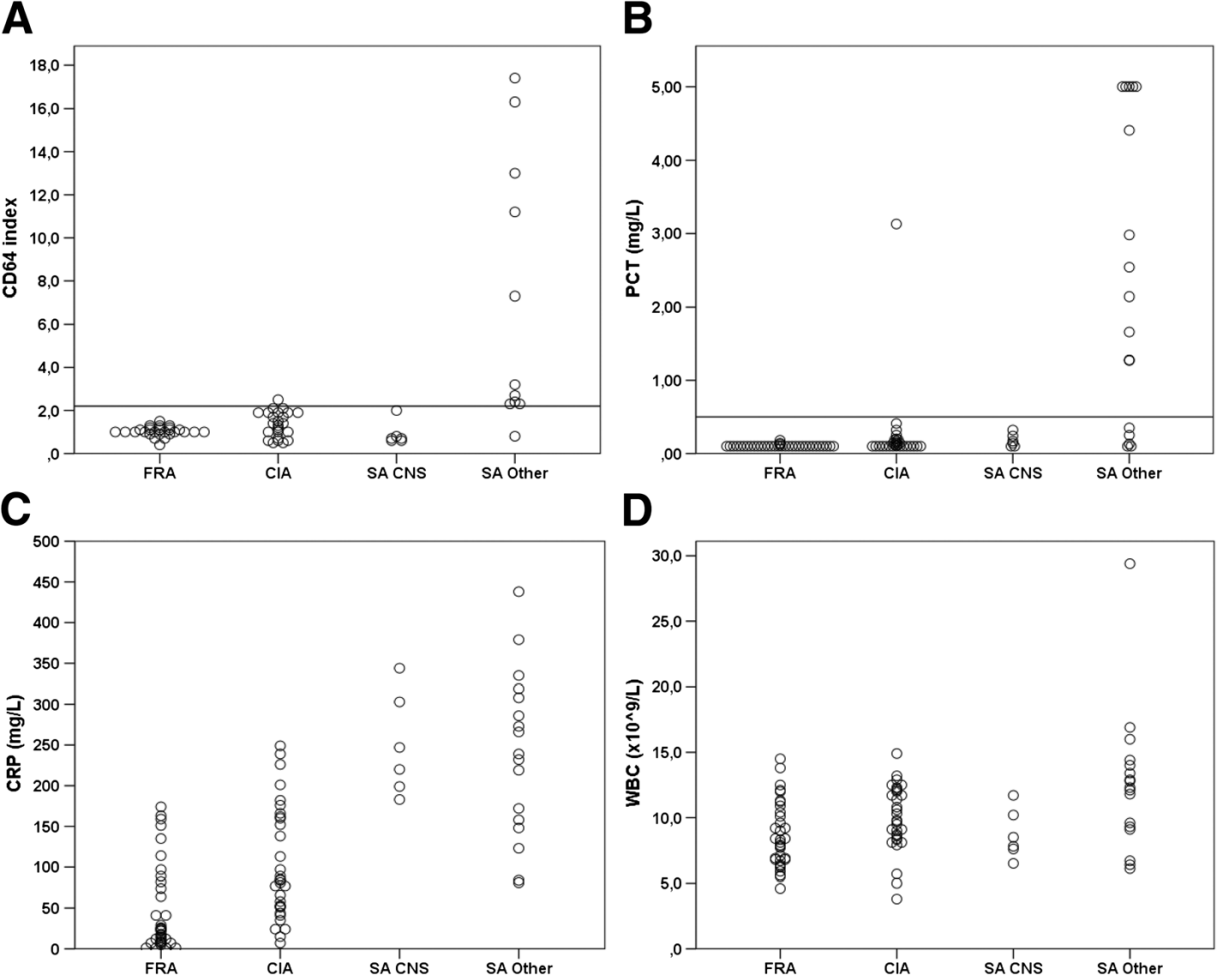
**Influence of low-pathogenicity microbial etiology.** Serum concentrations of CD64 (**A**), procalcitonin (**B**), CRP (**C**) and white blood count (**D**) among patients with flare of rheumatic arthritis (FRA), crystal-induced arthritis (CIA), septic arthritis caused by coagulase negative staphylococci (SA CNS) and septic arthritis with other microbiological agent (SA other). Horizontal lines indicate cut-off values for CD64 (**A**) and PCT (**B**), derived from ROC curve analysis. PCT values above 5 mg/L are plotted as 5 mg/L.

Subgroup analysis was conducted to evaluate the influence of the causative agent being coagulase negative staphylococci (CNS) on the discriminatory power of the inflammatory markers. When this patient group was excluded from analysis CD64 showed the greatest AUC of 0.92 (0.78-1.00), followed by CRP (AUC 0.91 (0.84-0.98)) and PCT (AUC 0.90 (0.79-1.00)). It appeared that excluding SA caused by CNS significantly improved the diagnostic reliability of CD64 and PCT (p = 0.001 and p = 0.01 respectively), while there were no statistically significant differences in the CRP or WBC values between SA caused by CNS versus other microbial etiology (p = 0.81 and p = 0.06 respectively)(Figure 2).

**Figure 3:**
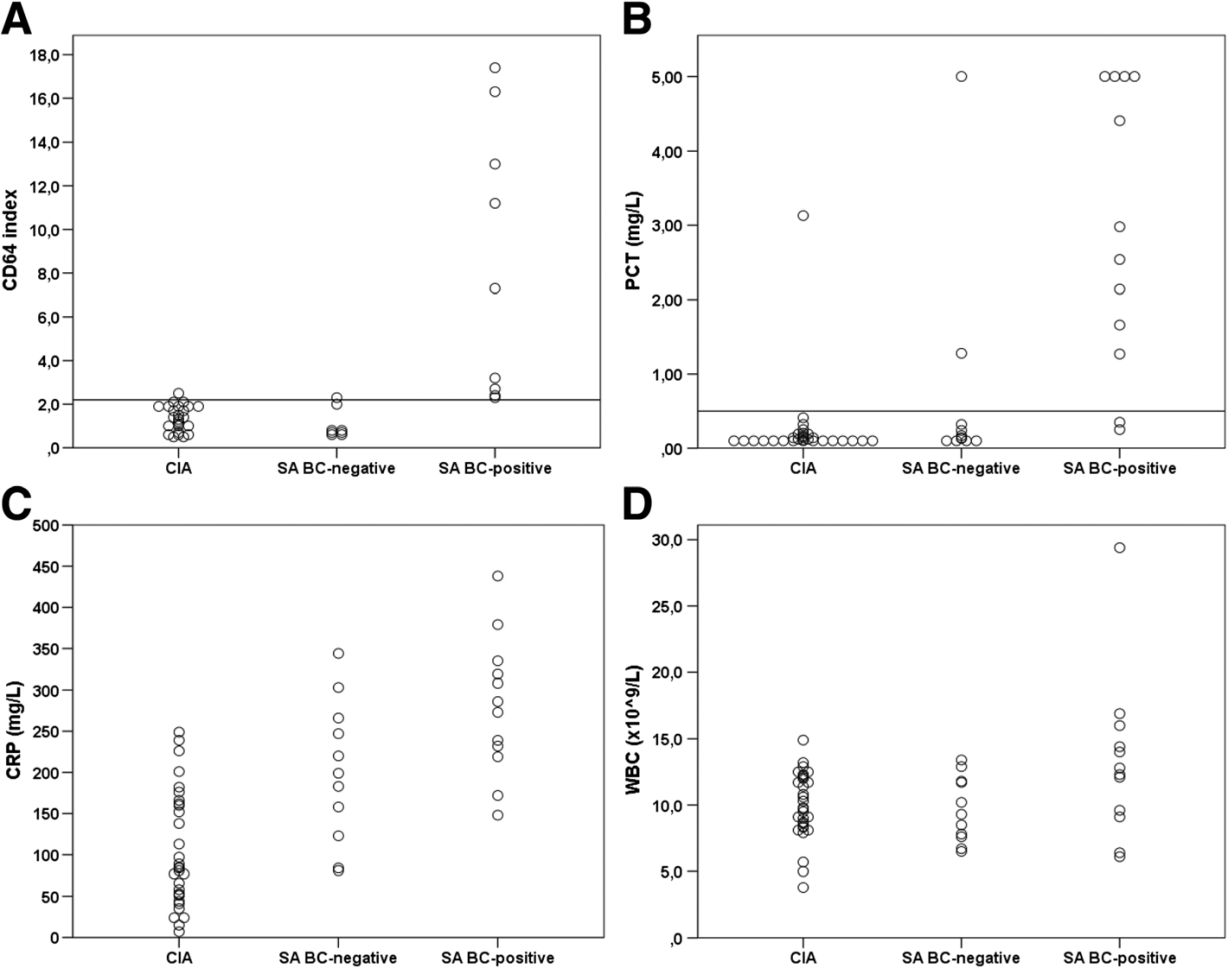
**Effect of localized versus systemic infection.** Serum concentrations of CD64 (**A**), procalcitonin (**B**) ), CRP (**C**) and white blood count (**D**) among patients with crystal-induced arthritis (CIA), septic arthritis with positive blood culture (SA BC-positive) and septic arthritis with negative blood culture (SA BC-negative). Horizontal lines indicate cut-off values for CD64 (**A**) and PCT (**B**), derived from ROC curve analysis. PCT values above 5 mg/L are plotted as 5 mg/L.

We further investigated the effect of septic arthritis with concurrent bacteremia versus local joint infection only. Figure 3 illustrates that with the CD64 index there was an almost complete segregation of the two groups, and a tendency towards the same concerning PCT. The values for CD64 and PCT did not differ significantly between patients with native or prostethic joints (p = 0.77 and p = 0.5 respectively).

## Discussion

The search for better diagnostic tools for identifying infectious disease is continuously ongoing. CD64 has previously been reported to, more reliably than CRP, differentiate systemic infections from rheumatic disease [8], surgical trauma [10] and viral infection [11]. To our knowledge, this is the first report on the use of CD64 in discerning septic arthritis from inflammatory joint disease. Doi et al. investigated the use of CD64 in patients with local inflammatory disease, including 22 patients with crystal induced arthritis [12]. They found low values for CD64 in all patients with CIA, but they did not perform comparison to patients with infectious arthritis.

We found CD64 to have comparable characteristics to procalcitonin. Both methods display a high specificity of 98%, and can be useful as a rule-in marker. However, their poor sensitivities make it difficult to rule out infectious disease. WBC and ESR were generally unhelpful, while CRP proved to have the best diagnostic accuracy as measured by AUC.

The most striking characteristic for CD64 and PCT in the present study was their apparent unresponsiveness to local infection. The subgroup of septic arthritis patients with positive blood cultures all had CD64 values above cut-off. Patients with negative blood cultures, on the other hand, had almost exclusively values below cut-off, and were indistinguishable from patients with crystal induced arthritis. Subgroup analysis in group 2 (UTI) for patients with and without and bacteremia, did not show the same segregation. This can partly be explained by the patient selection, since systemic affection (fever) was one of the inclusion criteria for this group. Our results indicate that CD64 and PCT predominantly measure systemic effects, and are less useful in identifying localized infections. This is in accordance with several previous reports on PCT and septic arthritis [13-15].

The cut-off value of 2.2 for CD64 correlates well with other studies employing Leuko64-kit [16-18]. This yielded a sensitivity of 59% and specificity of 98%. This is lower than a cumulative sensitivity of 79% and specificity of 91% found in a recent meta-analysis [4], although this meta-analysis mainly consisted of systemic infections. A novel meta-analysis by Li et al. examined 26 studies, and calculated a pooled sensitivity and specificity of 76% and 85%, respectively. The subgroup consisting of proven culture-verified infections, however, displayed a sensitivity of 78% and a specificity of 91% [19]. Tanaka et al. examined CD64 in local musculoskeletal infections, finding comparable diagnostic accuracy to our results with a sensitivity of 66% and a specificity of 96% [20].

The lower sensitivity for infection in our material compared to several previous studies may partly be explained by the high share in the present study of bacteria of low virulence, generating little inflammatory response. Accordingly, the exclusion of coagulase negative staphylococci from the analysis yielded significantly better diagnostic accuracy, and provided CD64 with the greatest AUC.

Furthermore, some studies have indicated a trend towards lower CD64 response to gram-positive bacteria than to gram-negatives [8], and the microbial flora in our cohort comprised mainly gram-positive bacteria. The varied microbial pathogenicity and the inclusion of prosthetic joint-infections impose difficulties interpreting the results, but are likely to reflect the cases encountered in hospital practice.

The use of culture as a gold standard for diagnosing septic arthritis is hampered by its lack of sensitivity, especially when antibiotics have been administered prior to admission. Inevitably, the etiology will remain elusive in some cases of acute arthritis, and the number of false negative cultures in the present study is accordingly unknown. The effect of excluding nine acute arthritis-cases due to uncertain final diagnosis is difficult to predict, but could potentially influence the calculated diagnostic accuracy of the inflammatory markers. The study is further limited by the lack of CD64-measurement in a subset of the inclusions.

CD64 has a short turnaround-time (1–2 hours) and is easily measured with flow cytometry. It has a quicker physiological response than CRP and SR, and cost is comparable to PCT. Their poor sensitivity in local infections, however, makes them unlikely to replace the current inflammatory markers used in hospital practice. CD64 was measured occasionally, but not systematically, in joint fluid specimens. Without exception the values were higher than their corresponding blood values, but the numbers were too small for statistical analysis. It is feasible that direct measurement of CD64 in joint fluid could prove more sensitive for local infection, and further studies are warranted to explore this possibility.

## Conclusions

CD64 and PCT are highly specific for infectious disease, including septic arthritis, and can be useful as rule-in markers. Their poor sensitivity in local infections, however, makes them unlikely to replace the current inflammatory markers used in hospital practice.

## Competing interests

The study has been fully financed by the Department of Medicine, Haukeland University Hospital. The authors report no conflicts of interests.

## Authors’ contributions

OO participated in the design of the study, inclusion of cases, performed the statistical analysis and drafted the manuscript. BS participated in the design of the study, inclusion of cases and helped to draft the manuscript. AKH included the cases for group 3, and revised the manuscript. NL conceived of the study, participated in its design and helped to revise the manuscript. All authors read and approved the final manuscript.

## Pre-publication history

The pre-publication history for this paper can be accessed here:

http://www.biomedcentral.com/1471-2334/13/278/prepub
